# Innate Immune Evasion of Lyme Disease Pathogen Drives Alzheimer-Like Pathology

**DOI:** 10.21203/rs.3.rs-8804079/v1

**Published:** 2026-03-18

**Authors:** Karita Haapasalo, Lilith Heiland, Deepak Kumar Vijaya Kumar, Pavel Uvarov, Alexander Moir, Anna Maaser-Hecker, Xun Wang, Eeva Juselius, Shahan Syed, Antti Tuhkala, Tommi Kajander, Maija Lappalainen, Jukka Hytönen, Markku Varjosalo, Doo Yeon Kim, Roger Kamm, Taru Meri, Se Hoon Choi, Rudolph Tanzi

**Affiliations:** University of Helsinki; University of Helsinki; Massachusetts General Hospital; University of Helsinki; Massachusetts General Hospital and Harvard Medical School; Massachusetts General Hospital and Harvard Medical School; Massachusetts Institute of Technology; University of Helsinki; University of Helsinki; University of Helsinki; University of Helsinki; Helsinki University Hospital; Turku University Hospital; Institute of Biotechnology, Helsinki Institute of Life Science HiLIFE, P.O Box 56, 00014 University of Helsinki, Helsinki, Finland; Harvard Medical School; Massachusetts Institute of Technology; University of Helsinki; Massachusetts General Hospital, Harvard Medical School; Massachusetts General Hospital

## Abstract

The amyloid β (Aβ) peptide is the main component of amyloid plaques in Alzheimer’s disease (AD). Growing evidence has pointed to a role for Aβ as an antimicrobial peptide (AMP). However, the interactions of Aβ with neurotropic pathogens and host evasion strategies have remained largely unexplored. Using quantitative proteomic analysis of patient cerebrospinal fluid (CSF), advanced biochemical methods, and four different 3D brain models, ranging from blood-brain barrier (BBB) microfluidic systems to 3D neurovascular networks, we show that Lyme neuroborreliosis (LNB) Borrelia spp. induce molecular and immunological alterations in the central nervous system (CNS) that resemble key pathological features of AD. These include upregulation of the complement cascade and a decrease in CSF Aβ levels. By assessing the antimicrobial action of Aβ against Borrelia spp., we demonstrate that Aβ acts as a pre-opsonin by promoting complement activation on microbial surfaces. We also show that LNB Borrelia spp. exhibit unique survival strategies that reduce Aβ binding and block oligomerization, while halting complement attack by recruiting complement regulator factor H. This facilitates bacterial adhesion to the BBB, and modulation of glial and cytokine responses, fostering CNS invasion. Our findings reveal a previously unrecognized mechanism of bacterial immune escape spanning the entire invasion pathway from the BBB to neuronal compartments, demonstrating that LNB Borrelia spp. evade Aβ-mediated antimicrobial action by interfering with opsonization and oligomerization of the peptide. Collectively, these findings provide a direct mechanistic link between pathogen immune evasion, Aβ dynamics, and neuroinflammatory cascades, advancing our understanding of infection-induced neuropathology, offering insights into novel potential therapeutic targets for AD and neuroborreliosis.

Lyme neuroborreliosis (LNB) is an invasive infection of the central nervous system (CNS) caused by three different genospecies of *Borrelia*, *B. burgdorferi, B. afzelii*, and *B. garinii* (*Borrelia* spp.) transmitted to humans by blood-feeding *Ixodes* ticks^1^. The most common sign of *Borrelia* infection is a ring-like rash called erythema migrans that develops around the tick bite. In 10–15% of cases, the bacteria migrate through the host tissues and reach the CNS within 2 weeks, leading to chronic neurological LNB^2^. Chronic infections with LNB *Borrelia* spp. are increasingly linked to long-term neurological disorders by numerous clinical, epidemiological, and mechanistic studies, but the cellular and molecular pathways driving this neuroinflammation remain unclear. Emerging evidence suggests that inter-organ communication between multiple bacterial species and the brain through the nose-brain, lung-brain, and gut-brain axes have been suggested^3,4^. The pathogenesis of LNB is different, involving direct contact between the bacterium and the CNS. LNB bacteria induce neuronal damage through immune-mediated neuroinflammation and, over time, may result in long-term neurodegenerative disorders.

The complement system is a key component of host innate immunity, constantly monitoring the bloodstream for microbial intruders. It initiates through three main pathways: classical, lectin, and the alternative pathways. In the alternative pathway (AP), an activated fragment of complement component, C3b, binds and opsonizes microbes for phagocytosis. At the same time, the enzyme C3b convertase (C3bBb) cleaves C3 into more C3b, enhancing the antimicrobial response. While AP spontaneously generates opsonizing C3b proteins on biological surfaces, the presence of AP regulator Factor H (FH) prevents unintended harmful effects on the host ^5–8^. FH binds to C3b on healthy host cell surfaces, inhibits C3 convertase formation, causing its decay. FH also functions as a cofactor for factor I to degrade C3b into inactive fragments. *Borrelia* spp. are masters of immune evasion and have evolved multiple ways to evade the complement system. The binding of FH by *Borrelia* spp. surface molecules is one of the best-characterized mechanisms to avoid attack by the AP. These include several proteins from the OspE-related family by LNB-causing *Borrelia*^9^ species, and FhbA expressed by several *Borrelia* species causing Relapsing fever (RF) ^10^. Similarly to LNB, RF may cause neurological complications, but these differ from those seen in LNB. LNB is characterized by active colonization of the CNS, persistent neuroinflammation, and neuropsychiatric or dementia-like manifestations, whereas CNS symptoms in RF are rare and are primarily driven by systemic inflammation and immune responses to acute infection ^11^.

Amyloid-β protein (Aβ) is an antimicrobial peptide, which is part of the brain’s innate immune defense against invading microbes. Aβ entraps a wide range of microbes in a protease-resistant fibrillar structure ^12–14^. Recent studies reveal that the antimicrobial potency of Aβ is dramatically amplified through functional synergy with human amylin, achieving far greater efficacy than either peptide alone ^15^. The accumulation of Aβ as amyloid plaques is also one of the main pathological hallmarks of Alzheimer’s disease (AD). Here, we assessed whether interactions between *Borrelia* spp., Aβ, and the immune system contribute to pathological changes in the brain that may underlie infection-induced neuroinflammation underscoring the role of Aβ as an antimicrobial peptide and mediator of immune responses linked to AD pathology. Proteomic analyses of CSF from LNB patients revealed heightened complement activation, cytokine dysregulation and reduced Aβ levels. Using human 3D neuronal and neurovascular models, we demonstrate how LNB *Borrelia* spp. manipulate host innate immune responses to invade the CNS. By comparing LNB and RF *Borrelia* species we show that Aβ targets and oligomerizes on RF *Borrelia* immune evasion molecules, reducing viability and increasing phagocytosis, while LNB *Borrelia* actively evade Aβ binding and oligomerization, enabling attachment to the BBB, and immune evasion. These findings strongly suggest that these infection-mediated immune responses, which enable bacterial persistence, can trigger or exacerbate Aβ pathology.

## Results

### Proteomic analysis of CSF from LNB patients reveals AD-like pathology

**Figure 1:**
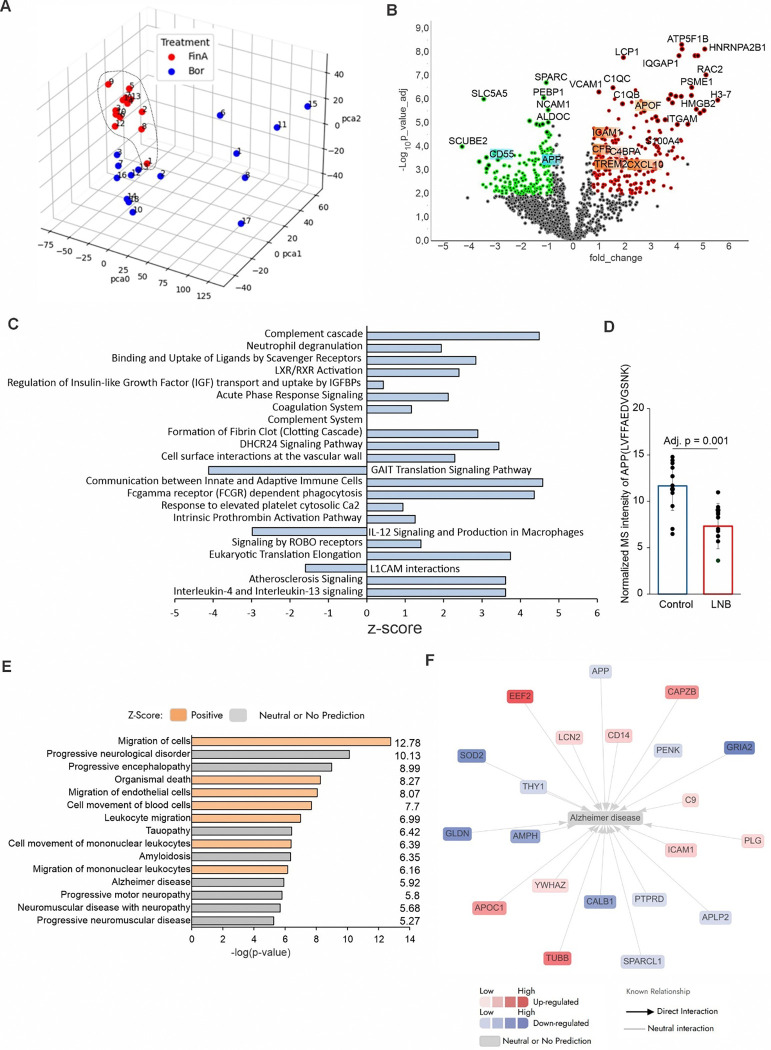
Mass spectrometry-based proteomic analysis of LNB CSF proteome identifies markers and signaling pathways that overlap with AD pathology **A,** Principal component analysis (PCA) of proteomic data separates LNB patient CSF samples (n=15) into two clusters distinct from healthy adult controls (n=13). **B,** Volcano plot highlighting differentially expressed proteins in LNB CNF samples compared to adult controls, with 276 upregulated proteins shown in red and 220 downregulated proteins in green (adjusted p-value < 0.05, fold change Z 1.5). **C,** Pathway enrichment analysis highlights significant complement cascade activation alongside cytokine responses. **D,** Normalized intensity of APP peptide levels showing reduction in LNB samples compared to controls. **E,** Disease pathway analysis ranks AD highest among neurological disorders affected by LNB. **F,** Network map of AD-associated proteins that are significantly affected in LNB, with upregulated (red) and downregulated (blue) nodes indicating direct interactions (arrows) and neutral predictions (gray).

Principal component analysis (PCA) showed two distinct clusters in LNB patient CSF proteomic data and a third separate cluster in proteomic data from controls (Fig. 1A). Differential proteomics analysis identified 276 upregulated proteins including CXCL10, TREM2, C1QC, C1QB, ACTG1, FCGPB and 220 downregulated proteins such as APP, CNTN1, LYNX1, NCAM1, THY1 consistent with prior LNB proteomic studies (Fig. 1B) ^16,17^. Pathway enrichment of the CSF proteome highlighted heightened activation of the complement cascade and pronounced cytokine responses, with elevated IL-12, IL-4 and IL-13 as well as IL-6 and IL-8 emerged from pathway interference levels suggesting efficient complement-evasion strategies of this pathogen (Fig. 1C and Extended data excel file S1). Strikingly, levels of Aβ were markedly decreased in LNB CSF, suggesting enhanced Aβ oligomerization and deposition in the brain tissue, leading to impaired Aβ transcytosis across the blood brain barrier (BBB), both of which are characteristic features of AD pathology (Fig. 1D). Disease-association mapping using Ingenuity Pathway Analysis (IPA) linked the LNB CSF proteome to multiple neurological disorders, with AD-related gene sets. These proteomic shifts mapped to pathways governing cell migration and multiple neurological disorders, with the AD pathway ranking highest among the disease associations (Fig. 1E and F, Extended data Fig. S1). These findings suggest that molecular changes observed in LNB infection overlap with pathways implicated in AD pathology.

### LNB *Borrelia* spp. evade Aβ42 binding and antimicrobial activity

**Figure 2:**
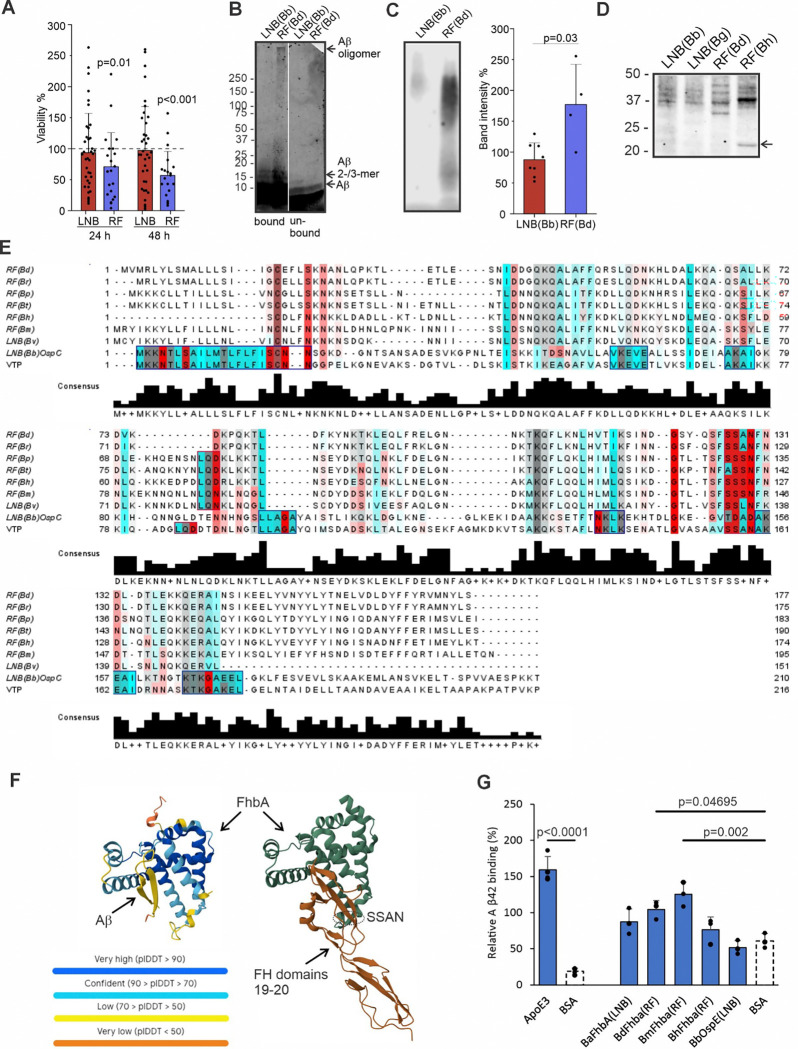
Target-specific binding of synthetic Aβ42 to microbes and microbial molecules. **A**, Viability (%) of *Borrelia* spp. incubated with 2 μM Aβ42, expressed relative to untreated controls. Data represent combined viability for two LNB *Borrelia* species, *B*. *burgdorferi* (Bb) and *B*. *garinii* (Bg), and two RF species, *B*. *duttonii* (Bd) and *B. hermsii*(Bh) (n = 21–38). **B**, Western blot (WB) showing surface-bound Aβ42 oligomers and 2-/3-mers on *Borrelia* spp., following Aβ42 incubation and detection with anti-Aβ antibody. **C-D**, Representative images of (C) semi-native PAGE and (D) SDS-PAGE followed by WB of the isolated *Borrelia*surface proteins (17 μg) pre-incubated with 2 μM Aβ42 and detection with anti-Aβ antibody. WB signal from semi-native PAGE is quantified relative to mean intensity per experiment (n = 3–8), with significance assessed by the Mann-Whitney test. The ~23 kDa RF (*B*. *hermsii*, Bh) band (arrow) was identified as variable tick protein (VTP) by mass spectrometry (UniProtKB/Swiss-Prot: Q3L772). **E**, Multiple sequence alignment of *B*. *hermsii* VTP, FhbA proteins, and OspC *Borrelia*surface proteins created with Jalview (accession numbers: FhbAs - Bd, *B*. *duttonii* W6TXL9; Br, *B*. *recurrentis* C1L349; Bp, *B*. *parkeri*D5GU46; Bt, *B*. *turicatae* B0L8C8; Bh, *B*. *hermsii*B5RLT5; Bm, *B*. *miyamotoi* A0A075BUA1; Bv, *B*. *valaisiana* C0R979; OspC, Q07337). Sequence similarities are boxed; small/polar residues in red, hydrophobic in blue (lighter shades for partial similarity), gray for no shared properties. Bar height indicates residue conservation. **F**, AlphaFold 3 prediction of Aβ42 interactions with *B*. *miyamotoi* FhbA, alongside the published FhbA-FH domains 19–20 structure (PDB: 6ZH1). The SSAN sequence (FH-binding residues on FhbA) is circled. **G**, Aβ42 binding to *Borrelia* FhbA and OspE detected by ELISA. ApoE serves as a positive control; BSA serve as negative control. Binding is relative to the mean absorbance at 450 nm per assay (n=3–5), with significance assessed by one-way ANOVA followed by Dunnett’s multiple comparison test. Error bars represent standard deviations.

Since epidemiological data have not suggested a causal link between LNB and AD ^18^, we next set out to explore the biological mechanisms underlying the observed AD-like proteomic signatures in LNB CSF, focusing on *Borrelia*-Aβ interactions. To determine whether synthetic Aβ can directly affect bacterial viability, *Borrelia*bacteria were incubated with 2 μM of synthetic Aβ42 prior to culturing. The presence of Aβ42 resulted in a significant reduction in viability of RF *Borrelia*spp*. (B. duttonii* and *B. hermsii)* but not LNB *Borrelia*spp*. (B. burgdorferi* and *B. garinii)* at 24- and 48-hours post-treatment (Fig. 2A). Large Aβ42 oligomeric complexes (MW > 250 kDa) were detected by Western blot (WB) on the surface of RF *B. duttonii*, while mainly Aβ42 monomers, 2-, and 3-mers were found on LNB *B. burgdorferi* surfaces (Fig. 2B), suggesting that the reduced viability of the RF *Borrelia* spp. is caused by surface oligomerization of Aβ42.

To explore the target specificity of Aβ42 on the surface of RF and LNB *Borrelia* spp., the surface proteins from the bacteria were first extracted, followed by incubation with Aβ42. Low affinity interactions were analyzed by a semi-native PAGE (Fig. 2C and Extended data Fig. S2A) while high-affinity interactions between Aβ42 and surface proteins were detected using a standard SDS-PAGE (Fig 2D). The intensity of Aβ42 bound to RF *Borrelia* spp. surface proteins was significantly higher than Aβ42 bound to LNB surface proteins, suggesting multiple binding interactions and/or formation of Aβ oligomers (Fig. 2C and Extended data Fig. S2A). The WB showed a distinct Aβ42 binding pattern on RF *Borrelia* spp. surface proteins when compared to LNB surface proteins, with the strongest Aβ42 signal observed on RF *B. hermsii* proteins (Fig. 2D).

To investigate the key mediators of the interaction between RF *Borrelia* and Aβ42, mass spectrometry analysis of a prominent ~23 kDa band from RF *B. hermsii* was performed and identified variable tick protein (VTP), sharing 48% similarity with LNB *B. burgdorferi* outer surface protein C (OspC), a known complement evasion factor ^19^. Multiple sequence alignment of VTP with OspC and other *Borrelia* FH-binding proteins (FhbA) revealed a conserved SSAN sequence in FhbA’s hinge region across species ^10^ (Fig. 2E). FhbA is an important virulence factor responsible for binding complement regulator FH ^10^. Leveraging FhbA’s high-resolution structure in complex with FH domains 19–20, AlphaFold3 predictions indicated that the β-sheet structure of Aβ42 allows the peptide to bind the L-shaped cavity (α-helices 3, 8, 9) of FhbA. This could potentially lead to nucleation of amyloid aggregation on the bacterial surface while overlapping the hydrophobic pocket in α-helix 7 of FH (Fig. 2F)^10^.

Given that FhbA can function as a scaffold for amyloid aggregation, sequence variability in FH-binding proteins may dictate Aβ42 susceptibility. By testing recombinant FH-binding proteins from various *Borrelia* species, we observed Aβ42 binding to the FhbA domains of RF *B. miyamotoi* and *B. duttonii*, but not OspE from LNB *B. burgdorferi* (Fig. 2G). This differential binding, together with reduced RF *Borrelia* survival and enhanced surface oligomerization, underscores that the innate target specificity and antimicrobial efficacy of Aβ are dictated by specific *Borrelia* species. These findings also highlight the adept evasion tactics of various LNB strains.

### Aβ42 and factor H binding underlies species-specific differences in microbial innate immune evasion

**Figure 3:**
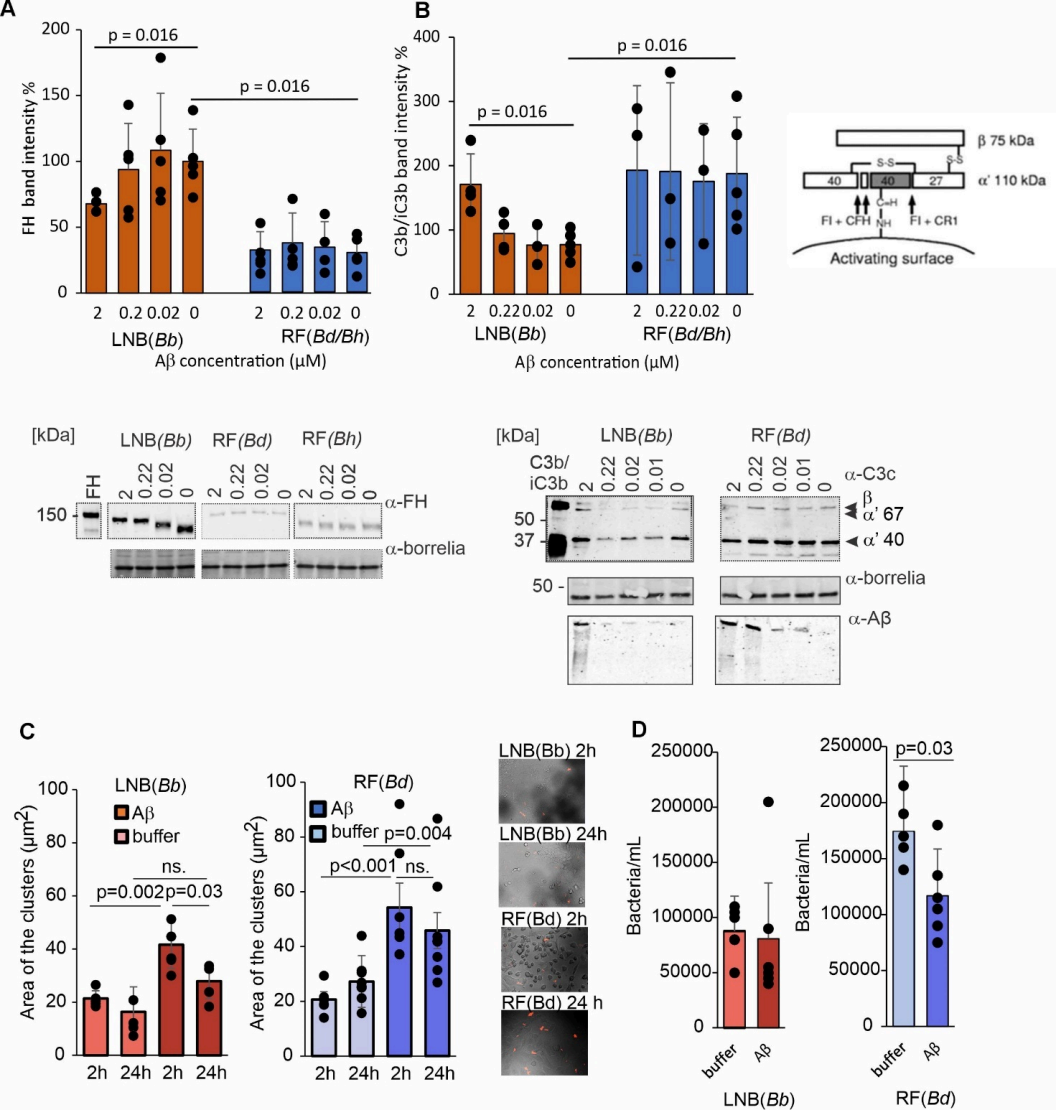
Aβ42 and Factor H (FH) Binding Underlie Species-Specific Differences in Microbial Complement and Phagocytosis Evasion, Favoring LNB Borrelia Survival. **A,** Quantification (top) of Western blots (bottom) detecting *Borrelia*spp. surface-bound FH in the presence of decreasing Aβ42 concentrations and 10% human serum (n=3). **B,** Schematic presentation of factor I (FI) mediated cleavage of C3b in the presence of factor H (CFH) cofactor (top right). Quantification (top left) of Western blots detecting C3b or iC3b (C3b/iC3b) cleavage fragments and a high molecular weight oligomer of Aβ on the stacking gel (bottom). A *Borrelia*-specific antibody was used as a loading control. **C,** Microglia-induced clustering of Aβ42/*Borrelia* spp. aggregates after 2 h and 24 h in SV40 microglia culture in the presence and absence of Aβ42 (n=6). Microscopy images showing the presence of Alexa-568-labelled *Borrelia* spp. aggregates in SV40 microglia. **D,** Phagocytosis of *Borrelia* spp. by SV40 microglia assessed by counting the remaining bacteria in the supernatant (n=6). Significances are calculated by Mann-Whitney U-test. Error bars show standard deviations.

To understand the complexity of RF *Borrelia* Aβ42 susceptibility and dual evasion of Aβ42 and complement by LNB *Borrelia* species in more physiological conditions, we next investigated whether Aβ42 binding to *Borrelia* FH-binding proteins alters complement resistance and phagocytic uptake. First, we tested complement evasion of *Borrelia* spp.in the presence of Aβ42 and normal human serum. Bacteria were pre-incubated with increasing concentrations of Aβ42 and then exposed to active complement present in human serum. Western blots of surface bound FH suggested significantly higher FH intensity on LNB *B. burgdorferi*than RF *Borrelia* spp. (Fig. 3A).However, when the concentration of Aβ42 increased (Fig. 3A and Extended data Fig. S2B), less FH was bound to LNB *B. burgdorferi,* suggesting competitive binding between FH and Aβ42 on the surface of *Borrelia*.

The intensity of iC3b was quantified using anti-C3c antibody, which detects both C3b and iC3b (C3b/iC3b), and was based on the presence of both 75 kDa β-chain (presence in both C3b and iC3b) and 68 kDa α-chain (present only in iC3b). The levels of iC3b increased in a Aβ42 concentration-dependent manner indicating that the deposited Aβ42 activates the complement (Fig. 3B and Extended data Fig. S2B). This is consistent with previous studies, in which Aβ aggregates have been shown to activate the complement system in the brain ^20^. However, in addition to the 68 α-chain, the presence of the 40 kDa α’-chain confirms C3b inactivation by proteolysis to iC3b thereby demonstrating the functionality of *Borrelia*surface-bound FH even at lower FH concentrations. In contrast, the limited binding of FH to RF *Borrelia* spp. did not affect C3b/iC3b levels suggesting limited complement evasion by this pathogen. These data suggest that Aβ42 binds to FH-binding proteins on *Borrelia* spp. On LNB *B. burgdorferi* stronger FH binding leads to competitive binding of Aβ42, which can potentially diminish *Borrelia* virulence by increasing complement deposition. However, this does not fully abolish the ability of LNB *Borrelia* to evade complement. In contrast, Aβ42 binding and oligomerization on RF *Borrelia* spp. did not show any competitive effect due to the weak binding of FH, making RF *Borrelia* spp. more susceptible to innate immune attack in the CNS.

Next, we investigated the effect of Aβ42 on microglia-mediated phagocytosis of *Borrelia* spp. For this purpose, we pre-incubated the bacteria with 2 μM of Aβ42 prior to exposing them to microglia. A rapid agglutination within 2 h was detected for both RF and LNB *Borrelia* spp. in the presence of Aβ42 when compared to bacteria incubated without Aβ42. After 24 h, however, only RF *Borrelia* bacteria were still agglutinated (Fig. 3C). Incubation of the bacteria in the presence of Aβ42 for 24 h (2 h vs. 24 h) resulted in a significant reduction of LNB *B. burgdorferi*agglutination while no significant change in RF *B. duttonii* agglutination was detected. This suggests that LNB *Borrelia* spp. can rapidly recover from Aβ42-mediated agglutination. The remaining *Borrelia* spp. were measured in the supernatant to assess microglial phagocytosis within 2 h (Fig. 3D). Bacterial concentrations of LNB *B. burgdorferi* remained the same in the presence vs. absence of Aβ42 while a significant reduction in RF *B. duttonii* concentrations was observed. These data indicate that binding and oligomerization of Aβ42 to the bacterial surface can enhance complement attack, microbial agglutination, and microglial phagocytosis, thereby promoting microbial clearance. However, LNB *Borrelia* spp. appear to possess the ability to recover from Aβ42-mediated antimicrobial effects and thereby evade microglial phagocytosis.

### LNB *B. burgdorferi* breaches the BBB and enhances permeability

**Figure 4:**
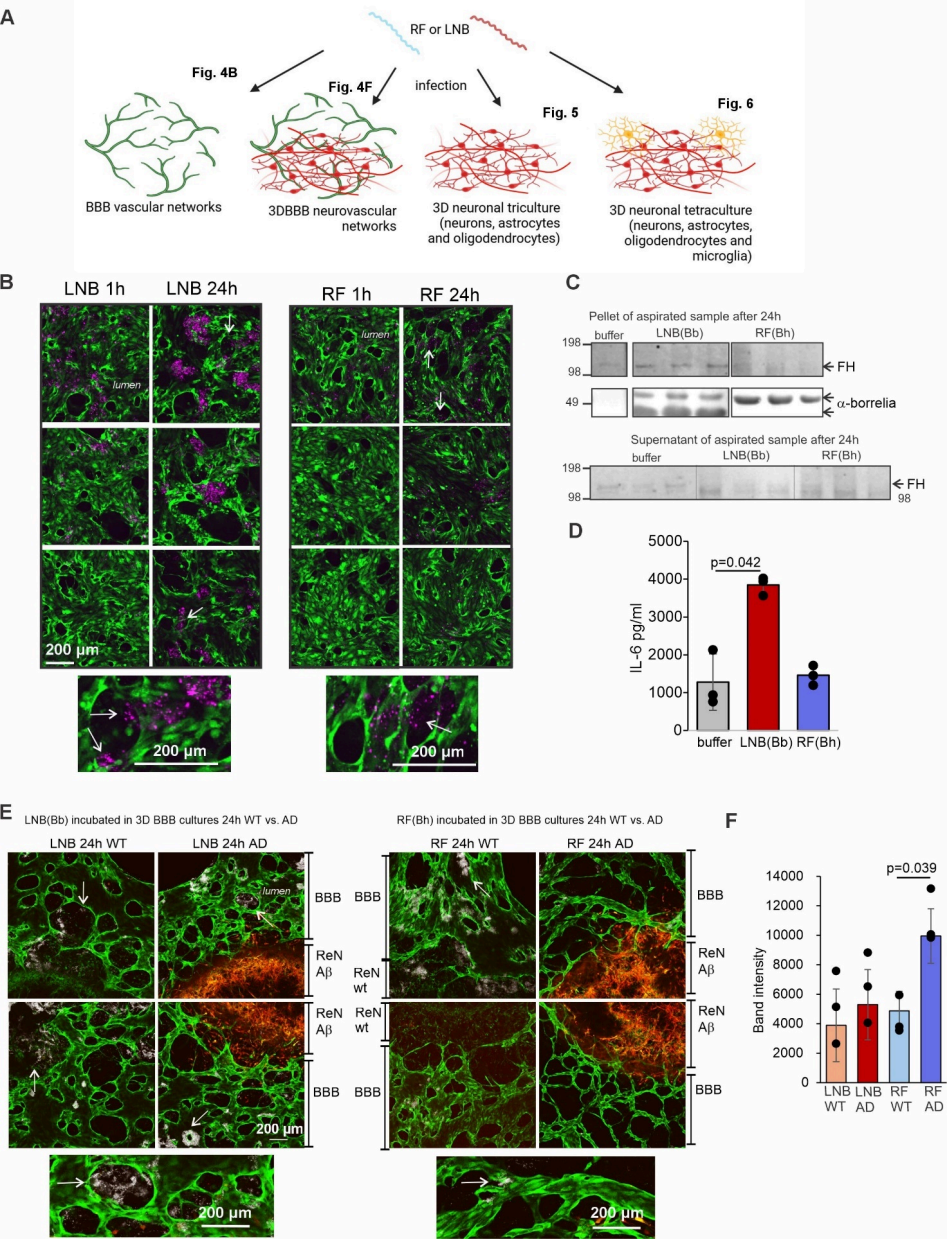
*Borrelia* spp. invasion and innate immune responses in BBB and BBB-3D neuronal cell cultures. **A,** Schematic presentation of the study workflow using a vascular (perfusable BBB model), 3D neurovascular (perfusable BBB vascular model combined with 3D triculture), 3D neuronal triculture (differentiated neurons, astrocytes and oligodendrocytes) and 3D neuronal tetraculture (differentiated neurons, astrocytes, and oligodendrocytes including iPSC-derived microglia) models. **B,** LNB *B. burgdorferi* and RF *B. hermsii* infections in a perfusable BBB culture. Movement, clustering, and BBB invasion (arrows) of *Borrelia* spp. are shown in the same region of interest at 1h and 24h time points. White arrows point to *Borrelia* spp. crossing the BBB. Moving from the lumen (lighter green central area, surrounded by a brighter green band corresponding to the BBB) to the black hollow areas located outside the lumen which represent the space outside the capillary **C,** Binding of 155 kDa FH (arrow) to LNB *B. burgdorferi* on isolated pellet from the BBB culture aspirate shown by WB. Detection of *Borrelia* spp. surface proteins by anti-borrelia (α-borrelia) antibody is used as a loading control. Presence of FH in the aspirated supernatant is shown in the buffer control. **D,** A significant increase in IL-6 cytokine levels in the BBB aspirate infected with LNB *B. burgdorferi* after 24 h measured by MSD V-PLEX Cytokine Panel. **E,**Infection of 3DBBB (green) showing BBB invasion of *Borrelia* spp. in the wild type (WT, not labeled) culture (no Aβ overexpression) vs. AD culture (Aβ overexpression, red). White arrows point to *Borrelia* spp. crossing the BBB. **F,** Quantification of *Borrelia*spp. protein band intensities from the WB of the aspirate shows increased levels of RF *B. hermsii* in the AD culture vs. WT culture indicating reduced attachment to the vasculature. Value (n=3) is the number of microfluidic devices used for each condition**.** Significances are calculated by one-way ANOVA with Dunnett’s multiple comparison post-hoc test. Error bars show standard deviations.

To further elucidate the infection mechanisms of neurotropic pathogens *in vitro*,we first exposed self-assembled, vascularized BBB networks-comprising primary human brain endothelial cells, pericytes, and astrocytes,to *Borrelia* spp. (Fig. 4A). We observed that LNB *B. burgdorferi* actively migrates and clusters within the vascular lumen before penetrating the BBB. In contrast, RF *B. hermsii* displays a more scattered distribution after 24 hours of incubation (Fig. 4B, and Extended data Fig. S3). The aspirated sample was centrifuged to separate bacteria from the supernatant. The pellet of LNB *B. burgdorferi* revealed FH deposition, while no clear FH bands could be detected from the bacterial supernatant and from the RF *B. hermsii* samples (Fig. 4C). The bacteria-free control confirmed FH presence in the supernatant, validating FH binding to the bacteria. These findings align with the serum exposure findings, reinforcing LNB’s evasion tactics. LNB *B. burgdorferi* also markedly elevated IL-6 cytokine levels (Fig. 4D), driving a potent inflammatory response that likely compromises BBB integrity. In contrast, RF *B. hermsii* induces a weaker inflammatory reaction, suggesting limited impact on endothelial function and BBB stability.

To further investigate the role of Aβ within the BBB microenvironment, we infected BBB networks, cultured with ReN neural progenitor cell line expressing Familial AD-associated APP mutations (Swedish K670N/M671L and London V717I), as well as the PSEN1 ΔE9 mutation (ReNAβ) ^21^ (3DBBB). For a wild-type control, a ReN line expressing only fluorescent protein was co-cultured with BBB networks (ReNwt). Incubation with *Borrelia* spp. in the cultures led to active clustering of LNB *B. burgdorferi* within the vascular lumen and successful BBB invasion in both ReNwt and ReNAβ cultures (Fig. 4E and Extended data Fig. S4A). Conversely, RF *B. hermsii* invaded ReNwt cultures but were at reduced levels in ReNAβ cultures, indicating a protective effect of Aβ against this pathogen at the BBB. The reduction of RF *B. hermsii* attachment to the BBB was quantified by WB analysis of the aspirated samples (Fig. 4F and Fig. S4B). We also observed a significant increase in RF *B. hermsii* proteins in the aspirate from ReNAβ cultures as compared to the ReNwt cultures, reflecting reduced attachment of the bacterium in the vascular wall in the presence of Aβ. These data correlate with the microscopy data showing diminished RF *B. hermsii* adhesion to ReNAβ vascular surfaces versus greater attachment to ReNwt cultures.

### LNB *Borrelia* spp*.* are resistant to Aβ antimicrobial activity

Using the 3D neurovascular model, we demonstrated that LNB Borrelia exhibit a distinct clustering and invasion phenotype at the BBB as compared to RF Borrelia, together with Aβ resistance. To investigate the mechanism underlying Aβ-mediated neutralization of RF *Borrelia* spp. following BBB invasion, we infected 3D neuronal cultures directly with the bacteria. These ReN 3D tricultures (Fig. 4A) comprising of ~70% neurons, ~30% astrocytes, and ~1% oligodendrocytes serve as an *in vitro* model that mirror tissue-level infection of bacterial infection dynamics. The two 3D cultures derived from ReN cell lines overexpress GFP and harbor either wildtype APP (WT) (ReNwt) or APPSwedish together with the APP I47F and I45F mutations (ReNAβ), leading to increased Aβ overexpression and AD-type pathology ^22^.

**Figure 5:**
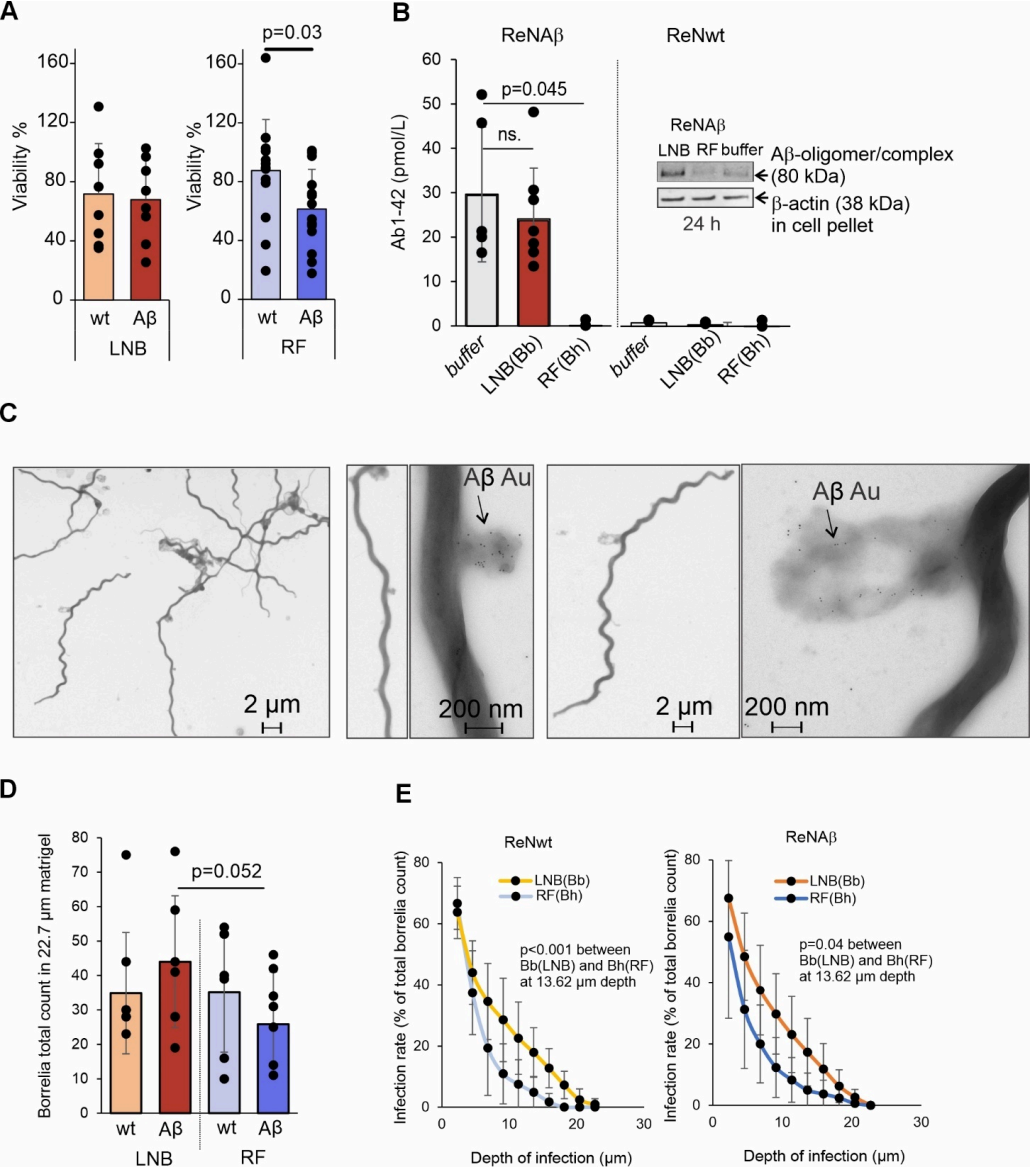
Effects of cellular Aβ on *Borrelia* spp. viability. **A,** Aβ over-expressing (Aβ) and wild type (wt) triculture supernatants affecting viability of LNB and RF *Borrelia spp.* after 24 h (n = 4). **B,** Significant reduction of soluble Aβ detected in the infected triculture supernatant in the presence of RF *Borrelia* spp. compared to a non-infected culture overexpressing Aβ (ReNAβ). No change was detected in the levels of soluble Aβ in the presence of LNB *Borrelia*. Trace amounts of Aβwas detected in the triculture supernatants taken from 3D culture expressing wild type APP (ReNwt) (n=3–7). Aβ oligomers detected by WB in the culture supernatant after removal of the bacteria by centrifugation and β-actin loading control detected in the extracted cell pellet (first lane) (n=1). **C,** Transmission electron microscopy (TEM) image of RF *B. hermsii* which was isolated from 3D triculture supernatant by centrifugation and labeled with anti-Aβ antibody coupled with gold nanoparticles. **D,** Bacterial total counts from a 22.7 μm Matrigel in 3D tricultures (n=4) **E,** Bacterial migration showing infection rate (depth of infection μM) of *Borrelia*spp. in (left) wt and (right) Aβ 3D tricultures. The bacteria were counted across 10 z-sections from the confocal microscopy images and the depth of infection was determined as % of bacteria present in lower sections relative to the total amount of bacteria in the image. The images (8 images/condition) are from two independent experiments that contains two biological replicates (n=4). Significances are calculated by Mann-Whitney U test (A, D, E) or one-way ANOVA supplemented with Dunnett’s test (B).

Exposing the bacteria to cellular Aβ by incubation with cell-culture supernatants significantly reduced the viability of RF *Borrelia* spp. In contrast, Aβ-containing supernatants had no effect on the viability of LNB *Borrelia* spp. (Fig 5A). To assess effects on Aβ oligomerization, the tricultures were incubated with *Borrelia* spp. and the concentration of soluble Aβ was measured in the supernatant. In cultures infected with the *Borrelia* spp., we observed a significant reduction in soluble Aβ, indicative of oligomer formation, in the culture supernatant in the presence of RF *B. hermsii*, but not LNB *B. burgdorferi* (Fig. 5B). The supernatant isolated from bacterial pellet after 24 h incubation revealed a trend toward reduction of an 80 kDa Aβ-containing band in the presence of RF *B. hermsii,* compared with LNB and the non-infected control, suggesting that the 80 kDa insoluble Aβ is attached to the microbe. Competitive binding between Aβ and FH and the increased binding of FH to LNB *Borrelia* spp. (Fig 3A and 4C)indicate that the reduction in viability is likely caused by both the direct toxic effects of Aβ oligomerization on the bacterial surface as well as reduced FH binding and complement deposition. Detailed visualization of Aβ on the bacterial surface by TEM imaging confirmed that Aβ deposits are indeed concentrated on disrupted areas of the bacterial membrane (Fig 5C).

To evaluate tissue invasion, we next compared the migration of RF and LNB *Borrelia* spp. in ReN 3D tricultures. We used confocal microscopy and counted the number of bacteria in the full stack and calculated the percentage of bacteria that migrated through Matrigel (Fig. 5D). Interestingly, the bacteria count of LNB *B. burgdorferi* revealed a trend toward increased levels in the presence of Aβ. In contrast, the counts of RF *B. hermsii* were significantly lower than those of LNB *B. burgdorferi* under the same conditions, consistent with the results in the 3D-BBB model (Fig. 4E and F). A comparison of the infection rate and depth of infection showed that LNB *B. burgdorferi* bacteria were localized more deeply in the Matrigel, and the bacteria counts were significantly higher than those for RF *B. hermsii* at a depth of 13.62 μm (Fig. 5E). Interestingly, the levels of Aβ in the tricultures had no effect on *Borrelia* invasion into the Matrigel.

### LNB and RF *Borrelia* differentially affect Aβ-driven innate immune responses

**Figure 6:**
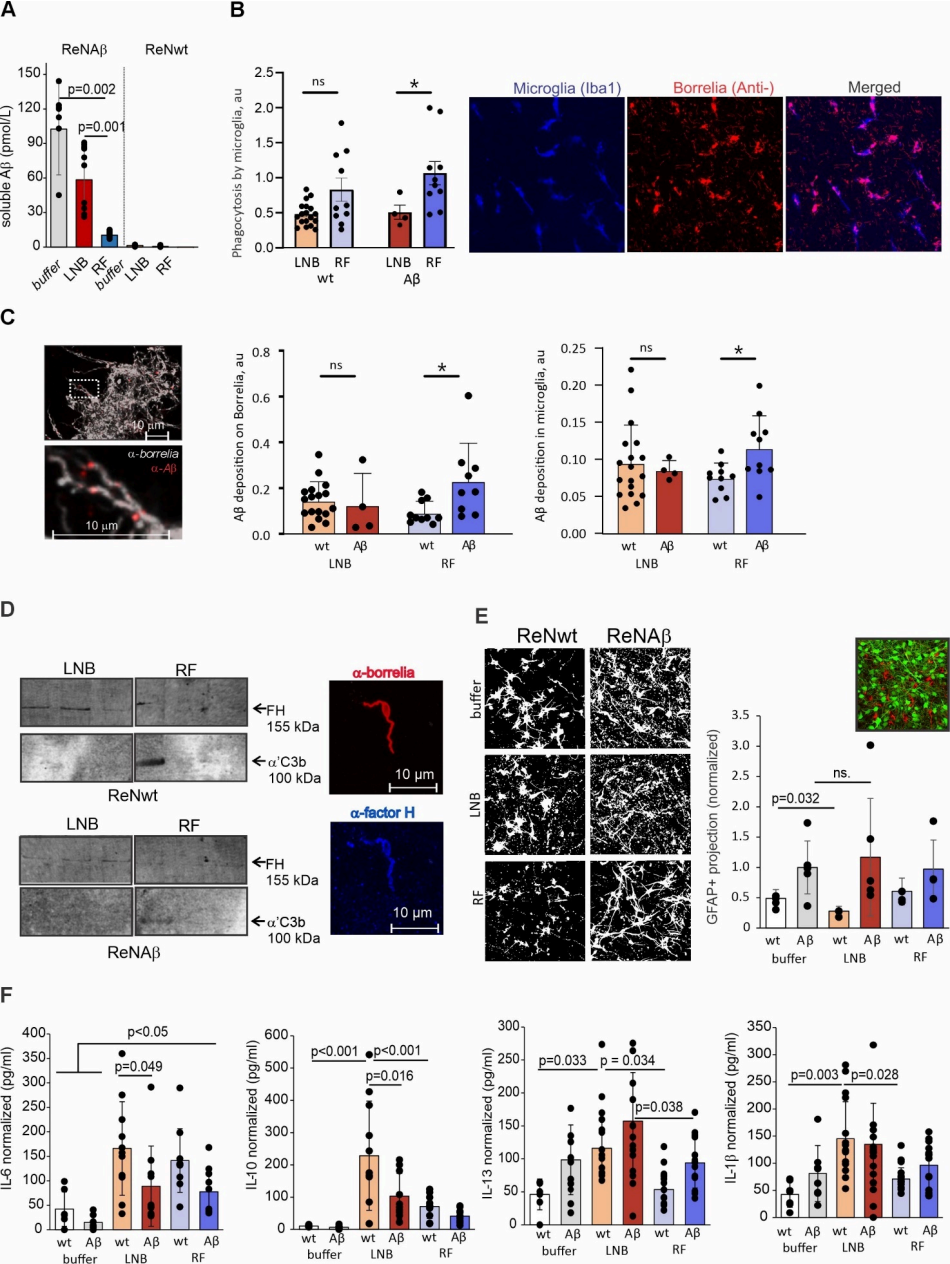
Aβ-driven microglial activation and cytokine profiles distinguish LNB from RF *Borrelia* infections in 3D tetracultures. **A,** Significant reduction of soluble Aβ was detected in the infected ReN 3D tetraculture supernatant in the presence of RF *Borrelia* spp. compared to a non-infected culture overexpressing Aβ (ReNAβ). No change detected in the levels of soluble Aβ in the presence of LNB *Borrelia*. Trace amounts of Aβ were detected in the tetraculture supernatants taken from 3D culture expressing wild-type APP (ReNwt). **B,** Phagocytosis of RF and LNB *Borrelia* was analyzed by confocal microscopy. Iba1 and anti-*Borrelia* antibodies were used to label microglia and *Borrelia*, respectively. The ratio between *Borrelia*spp. localized inside versus outside microglia was used as a measure of microglial phagocytosis. **C,** (left) Aβ binding to RF *B. hermsii* shown in the immunofluorescence image. (right) Aβ binding and phagocytosis is increased in RF *B. hermsii* infected cultures, indicating Aβ-mediated opsonophagocytosis. **D,** (left) WB quantification of the bacteria surface-bound FH and C3b on RF and LNB *Borrelia* spp. (right) FH (anti-factor H) detected on LNB *B. burgdorferi*surface (anti-Borrelia) by immunofluorescence microscopy. **E,** Astrogliosis was assessed by GFAP immunofluorescence staining in infected 3D tetracultures. Maximum projection images of GFAP-stained astrocytes in the control, RF, and LNB-infected 3D tetracultures are shown (left). Increased Aβ levels enhanced astrocyte activation, while LNB *B. burgdorferi* reduced astrogliosis in ReNwt but not in ReNAβ cultures. **F,** Cytokine levels in 3D tetraculture supernatants measured by MSD V-PLEX Cytokine Panel (see also Extended data Fig. S5). Experiments were repeated three times with at least two biological replicates (n = 6). Multiple comparison was performed using one-way ANOVA with Tukey’s or Dunnet’s multiple comparison post-hoc test or Kruskal-Wallis for comparing groups with non-normally distributed data. Differences between the two samples was calculated using Mann-Whitney U Test. Error bars show standard deviations.

To better understand how Aβ influences neuroinflammation during *Borrelia* infections, we next used advanced 3D tetracultures (Fig. 4A), which included iPSC-derived microglia along with neurons, astrocytes, and oligodendrocytes. This 3D tetraculture infection model offers several crucial advantages for recapitulating CNS biology and pathology. Because microglia play a crucial role in CNS inflammatory responses and are the main phagocytic cells eliminating both Aβ and invading pathogens, we supplemented the culture with iPSC-derived human microglia (10% of all cells, representing physiological levels of microglia in the frontal cortex) 6 days before infection. In line with the findings from the triculture model (Fig. 5B), soluble Aβ levels were significantly reduced only in the presence of RF *B. hermsii* (Fig. 6A), suggesting that in the tetraculture model, LNB exhibits resistance to Aβ-mediated effects.

Next, we assessed phagocytosis of RF and LNB *Borrelia* by microglia in the 3D tetracultures. ReNwt and ReNAβ 3D cultures were infected by RF and LNB *Borrelia* species and bacterial phagocytosis was analyzed using confocal microscopy (Fig. 6B). Microglia were labeled using antibodies against Iba1, while *Borrelia* spp. were labeled using anti-Borrelia antibodies. Colocalization of these two signals was used as a measure of phagocytosis (Fig. 6B). Specifically, for each 3D tetraculture type (ReNwt and ReNAβ) and each *Borrelia* group (RF, LNB), we quantified the ratio of *Borrelia* spp.localized inside versus outside microglia. We observed significantly higher phagocytosis of RF compared to LNB *B. burgdorferi*, but only in Aβ-expressing ReNAβ cultures. Immunofluorescence microscopy demonstrated colocalization of Aβ on the surface of RF *B. hermsii* (Fig. 6C), which is consistent with results using synthetic Aβ (Fig. 2B and 2C) and the supernatant and TEM images from the 3D triculture model (Fig. 5B and 5C). These findings suggest that Aβ binds and oligomerizes on the surface of RF *Borrelia* spp. while LNB *Borrelia* spp. evades Aβ binding and oligomerization. Moreover, significantly more Aβ phagocytosis was detected in RF *B. hermsii*-infected cultures as compared to WT cultures, suggesting Aβ-mediated opsonophagocytosis of this bacterium (Fig. 6C). Together with the observation of increased C3b deposition alongside elevated Aβ levels on the bacterial surface (Fig. 3B), these data suggest a novel role for Aβ as a pre-opsonin that facilitates complement activation and enhances microglia-mediated clearance.

FH and C3 were detected on *Borrelia* spp.surfaces isolated from 3D tetracultures. A trend toward reduced FH binding on RF *B. hermsii* compared to LNB *B. burgdoreferi* was observed (Fig. 6D), consistent with the results from bacteria incubated in the presence of 10% normal human serum (Fig. 3A) and in the BBB cultures (Fig. 4C). The presence of Aβ in ReNAβ cultures showed a similar trend toward reduced FH binding, suggesting that competitive binding between FH and Aβ on *Borrelia* surface increases C3b deposition (Fig. 3B), which may further amplify complement-mediated opsonophagocytosis. Next, we focused on astrogliosis, which is part of the inflammatory response activated in neuroborreliosis ^23^. Astrocytes are known to contribute to complement defense against infections by producing complement molecules^24^. First, we examined the levels of astrogliosis by GFAP staining and assessed astrocyte hypertrophy and branching in infected 3D tetracultures by immunofluorescence imaging (Fig. 6E). As expected, increased Aβ levels in the cultures led to enhanced astrogliosis. Surprisingly, in the presence of LNB *Borrelia*, astrocytes were less activated in ReNwt culture, but not in the ReNAβ culture. This suggests that LNB *Borrelia* may modulate the immune response, while Aβ may dampen the immunomodulatory effects of *Borrelia*.

The presence of both microglia and astrocytes in the infected 3D culture model provided a more comprehensive and physiologically relevant system for studying CNS immune responses during neuroborreliosis. Notably, the 3D triculture, which lacks one of the key cells, exhibited weaker cytokine responses following *Borrelia* infection (Extended data Fig. S5). *Borrelia* spp. infection of the 3D tetracultures led to an increase in IL-6 levels (Fig. 6F) as observed in the BBB cultures (Fig. 4D), including infection with RF *B.hermsii*. Interestingly, LNB *B. burgdorferi* infection increased the levels of cytokines (IL-10, IL-13, and IL-1β), which have previously been shown to mediate neuroinflammatory responses and neuropsychological dementia-like symptoms during *Borrelia* infection (Fig. 6F) ^25,26^. The *B. burgdorferi*-mediated IL-6 and IL-10 responses were significantly reduced in the presence of Aβ, further supporting a role for Aβ in microbial defense. In general, cytokine responses were less robust during RF *B. hermsii* infection, indicating LNB *B. burgdorferi* elicits stronger inflammatory and neurotoxic effects and CNS persistence*.* This was also supported by our viability assays (Fig. 2A, 3D, 5A), molecular-level data on Aβ binding and oligomerization (Fig. 2, 5B, 6A), and the effect of Aβ on complement activation (Fig. 3A and 3B) and phagocytosis (Fig. 3C and 6B). Collectively, these findings demonstrate efficient immune evasion of LNB *Borrelia*, which helps to explain certain neuropathological similarities with AD.

## Discussion

Cognitive impairment is a documented but rare complication of LNB ^27,28^. To date, the neuropathological features underlying these symptoms have not been fully defined. Although the dementia-like symptoms in LNB have been shown to be reversible in the most cases, persistent neurological symptoms characterized by episodes of seizures remain poorly understood^29^. Our proteomic analysis of LNB CSF samples revealed an overlap with pathways upregulated in AD involving the complement system, tauopathy and amyloidosis pathways. These findings suggest that LNB may trigger chronic inflammation, modulate Aβ processing and thereby contribute to cognitive dysfunction.

IL-6, IL-10, and IL-13 are key cytokines triggered by LNB infection, and these were consistently detected both in proteomic data from LNB patients and in our neuronal and neurovascular models. The presence of both IL-10 and IL-13 supports persistence of infection ^30^, whereas upregulation of IL-6 indicates increased BBB permeability, correlating with efficient crossing of the BBB by LNB *Borrelia* spp. Similarly, upregulation of IL-6 in AD is an established marker of BBB permeability, facilitating the leakage of serum factors into the brain that exacerbates neuroinflammation. Notably, neutralization of IL-6 has been shown to alleviate memory impairment in mouse models^31^. The precise mechanisms connecting these events remain to be elucidated. Interestingly, Aβ inhibited expression of *Borrelia*-induced cytokine expression in our 3D tetraculture model. This was not, however, sufficient to reduce the viability of LNB *B. burgdorferi*.

Here, we demonstrate that Aβ selectively targets evolutionarily conserved surface proteins needed for *Borrelia* spp. immune evasion, including factor H-binding proteins. Recent studies have identified microbial molecules that inhibit formation of amyloid assemblies ^32^, supporting our data on the target specific binding of Aβ, where interactions can either inhibit or promote Aβ oligomerization depending on the target molecule. Notably, this specificity likely explains how LNB *Borrelia* spp. escape this defense mechanism by interacting with Aβ without inducing Aβ oligomerization. Such selective binding and conserved interactions are hallmarks of innate immune molecules such as those in the complement system, providing further support for a role of Aβ as an AMP in the innate immune system. Our data also indicate that the AMP effect of Aβ extends to vascular sites as we observed Aβ to diminish the attachment RF *B. hermsii* to the vessel wall while LNB *B. burgdorferi* attachment remained unaffected.

Based on the TEM images, Aβ oligomers disrupt the membrane of RF *B. hermsii*, suggesting a mechanism of pore formation consistent with previous studies ^33^. However, whether membrane disruption, alone, is sufficient to eliminate the bacterium remains unclear. Importantly, our data show that Aβ competes with FH for binding on the *Borrelia* surface, which leads to C3b deposition, indicating that Aβ functions as a pre-opsonin by promoting complement activation against the pathogen. This competitive behaviour of Aβ displacing FH was evident on LNB *B. burgdorferi*, yet it did not lead to bacterial elimination. This may represent a deleterious cycle of persistent infection and sustained inflammation, which could exacerbate disease progression^34^.

Our study demonstrates the power of using human 3D neurovascular cultures in studying bacterial pathogenesis at the tissue level. *Borrelia* species are known to infect other mammals beyond humans, which has made animal models useful tools for studying these infections. However, *Borrelia* adapts to its host environment through antigenic and phase variation mechanisms ^35^. This form of host adaptation limits the usage of animal models, as they cannot fully recapitulate human infection ^36^. Our 3D models, however, provide a cellular microenvironment wherein the bacteria can directly interact with complex human immune components. As we demonstrate in this study, these interactions critically influence the survival and persistence versus clearance of the pathogen.

In conclusion, here, we describe the pathogenic pathway of *Borrelia* spp. from the BBB into the brain, integrating LNB patient data and four different cell culture models (Extended data Fig. S6). We show *Borrelia* spp. induce molecular and immunological alterations in the CNS resembling key pathological features of AD, including upregulation of the complement and a decrease in CSF Aβ levels owing to enhanced cerebral amyloid deposition. We also show that Aβ acts as a pre-opsonin by enhancing C3b deposition and complement activation on bacterial surfaces. We also demonstrate that LNB *Borrelia* spp. exhibit unique survival strategies by simultaneously reducing Aβ binding and oligomerization, while also halting complement attack by recruiting complement regulator factor H, fostering CNS invasion. Consequently, persistent *Borrelia* infection could drive chronic neuroinflammation while also promoting pathogenic Aβ deposition. Collectively, these findings offer novel insights into the physiological role of Aβ while providing a direct mechanistic link between pathogen immune evasion, Aβ deposition, and neuroinflammation. These findings should serve to not only advance our understanding of infection-induced neuropathology but also offer insights into novel potential therapeutic targets for both AD and neuroborreliosis.

## Methods

### CSF Samples

Patient samples for the present study were originally collected in a previously published LNB treatment study ^28^, where informed consent was obtained from every patient, and ethical approval was provided by the National Committee on Medical Research Ethics in Finland. The diagnosis of LNB patients was based on the criteria by EFNS ^37^, where IDEIA LNB test (Oxoid, Basingstoke, UK) was used to demonstrate intrathecal borrelia-specific antibody production. The control samples were collected at the Hospital District of Helsinki and Uusimaa from adult patients with no infection (n=13).

### Mass Spectrometry

#### Sample preparation

For cerebrospinal fluid analysis 10 μl for human CSF of each sample was taken and mixed with 15 μl 8 M urea and incubated on ice for 15 min followed by 10 min sonication in ice bath. After incubation leveled to 125 μl by adding 100 μl of 100 mM NH4HCO3 (AMBIC (#A6141, Sigma Aldrich)). Then all the samples were reduced with 5 mM TCEP (Tris(2-carboxyethyl)phosphine hydrochloride (#20490, Thermo Scientific)), alkylated with 10 mM iodoacetamide (#122271000, Acros Organics) at room temperature, and trypsin/LysC-digested at 37 °C for 16 hours using 4 μl Trypsin/LysC (0.5 μg/μl, V5071, Promega). Following digestion, peptides were quenched with 10% trifluoroacetic acid (TFA, #85049.051, VWR) and finally, the desalted with BioPureSPN PROTO 300 C18 Mini columns (#HUM S18V, Higgins Analytical) according to manufacturer’s instructions. The dried peptides were reconstituted in 30 μl Buffer A (0.1% (vol/vol) TFA and 1% (vol/vol) acetonitrile (ACN, #83640.320, VWR) in HPLC water, #10505904, Fisher Scientific).

#### Mass spectrometry and data analysis

For the DIA analysis the resuspended peptides were further diluted 1:4 in buffer A1 (1% formic acid in HPLC water). 20 μl was loaded into an Evotip (Evosep, Denmark) following manufacturer’s instructions. The samples were analyzed using the Evosep One liquid chromatography system coupled to a hybrid trapped ion mobility quadrupole TOF mass spectrometer (Bruker timsTOF Pro, Bruker Daltonics) ^38^ via a CaptiveSpray nano-electrospray ion source (Bruker Daltonics). An 8 cm × 150 μm column with 1.5 μm C18 beads (EV1109, Evosep) was used for peptide separation with the 60 samples per day methods (21 min gradient time). Mobile phases A and B were 0.1 % formic acid in water and 0.1 % formic acid in acetonitrile, respectively. The MS analysis was performed in the positive-ion mode with dia-PASEF method ^39^ with sample optimized data independent analysis (dia) scan parameters. We performed DDA in PASEF mode from a pooled sample to be able to adjust dia-PASEF parameters optimally. To perform sample specific dia-PASEF parameter adjustment the default dia-short-gradient acquisition methods was adjusted based on the sample specific DDA-PASEF run with the software “tims Control” (Bruker Daltonics). The following parameters were modified for each sample type: m/z range: 380.0 – 1065.0 Da; mass steps per cycle: 28; mean cycle time: 1.06 s. The ion mobility windows were set to best match the ion cloud density from the sample type specific DDA-runs. To analyze diaPASEF data, the raw data (.d) were processed with DIA-NN v2.0 ^40,41^ utilizing spectral library generated from the UniProt human proteome (UP000005640, downloaded 06.02.2025 as a FASTA file). During library generation following settings were used, fixed modifications: carbamidomethyl (C); variable modifications: acetyl (protein N-term), oxidation (M); enzyme:Trypsin/P; maximun missed cleavages: 1; mass accuracy fixed to 1.5e-05 (MS2) and 1.5e-05 (MS1); Fragment m/z set to 200–1800; peptide length set to 7–50; precursor m/z set to 300–1800; Precursor changes set to 2–4; protein inference not performed. All other settings were left to default.

### Bacterial Strains

LNB *B. burgdorferi* B31 (DSM 4680) and RF *B. hermsii* (DSM 5251) were ordered form DSMZ (German Collection of Microorganisms and Cell Cultures GmbH) or ATCC. LNB *B. garinii* SBK40, LD *B. burgdorferi* sensu stricto N40 were a kind gift from PhD MD Jukka Hytönen (University of Turku) and RF *B. duttonii* CR2A from PhD Adj. professor Taru Meri (University of Helsinki). *Borrelia* spp. were cultured in BSK-H complete medium (Barbour-Stoenner-Kelly medium; B8291, Merck Life Science) or BSK-H medium (1–10S02-I; BioConcept) supplemented with 6 % rabbit serum (R4505, Merck Life Science, Molsheim, France). *Borrelia* spp. were grown at 35 °C in 5 % CO2 and under non-shaking conditions. They were grown to maximally passage 10. The number of bacteria was assessed via dark field microscopy utilizing the Olympus U-DCW oil immersion darkfield condenser.

### Complement Activation Assay

Borrelia spp. Were pelleted at 3000 × g for 10 min and washed three times with 1x PBS at 16,000 × g for 2 min. They were diluted in a volume of 100 μL to 1×10^8 bacteria/mL and incubated for 4 h at 600 rpm, 35 °C, and 5 % CO2 in a 1:3 serial dilution of 2 μM Aβ42. *Borreli*a spp. were pelleted at 16,000 × g for 2 min and diluted in 100 μL of 10 % normal human serum provided in 1x PBS. After another 30 min incubation step with the same conditions, all tubes were moved on ice and 10 mM of EDTA were added. The Borrelia spp. were pelleted at 16,000 × g for 2 min and washed three times with 1x PBS. After this 90 μL of 0.1 M glycine-HCL (pH 2.3) were added and the bacteria were pelleted again. The supernatant was then combined with 100 mM of Tris (pH 8) and the samples were used for western blotting.

### Polyacrylamide Gel Electrophoresis

PAGEs were run, both in reducing and semi-reducing conditions. SDS-PAGEs, samples were preheated with 4x LDS Sample Buffer (B0007, Thermo Fisher Scientific) with or without 10 % of the Sample Reducing Agent (B0009, Thermo Fisher Scientific) for 5 minutes at 95 °C for reducing conditions or at 37 °C without the Sample Reducing Agent for semi-reducing conditions. Also, a 4x sample buffer (0.25 M Tris base, 0.28 M SDS, 40 % glycerol) was used with 65 °C adding the Sample Reducing Agent for reducing conditions.

### Western Blotting

Samples were run on the Mini-PROTEAN^®^ TGX Stain-free TM Precast Gel (4568096, Bio-Rad) for 1 h at 100 V with the Precision Plus Protein^™^ Kaleidoscope^™^ ladder (1610375, Bio-Rad). TGS-buffer (1610732, Bio-Rad) was used under reducing conditions and TG-buffer (1610734, Bio-Rad) under semi-reducing conditions. Gels were imaged with the ChemiDoc XRS+ (Bio-Rad, Hercules) and the proteins transferred onto a nitrocellulose membrane utilizing the semi-dry western blotting machine (Bio-Rad) for 6 minutes at 15 V for each gel. Unspecific binding sites were blocked by incubating the membrane for either 2 hours at room temperature or overnight at 4 °C in 3 % fat-free milk in PBS under shaking conditions. Primary antibodies were diluted to 0.25 μg/mL in 0.3 % PBS-milk and dilutions were stored at −20 °C and reused five times by applying 0.1 μg/mL fresh antibody (Table 1). The primary antibody was incubated for 1 hour at room temperature or overnight at 4 °C with the membrane under constant shaking. The membrane was washed three times with 1x PBS, interrupted by 10-minute shaking steps at room temperature. The secondary antibody was diluted to 0.1 μg/mL in 0.3 % PBS-milk and incubated with the membrane for 1 hour at room temperature under shaking conditions and washed three times with PBS again. The western blot was imaged with the Odyssey CLx Imaging System (LI-COR Biosciences). Antibodies that were used in different combinations are: goat anti-factor H (341276, Calbiochem), mouse anti-Aβ (MABN10 Millipore), rabbit anti- Aβ (702254, Invitrogen), rabbit anti-C3c rabbit (15338624 Behring), rabbit anti-Borrelia burgdorferi (ABX48–1KC, Merck Millipore) and corresponding secondary antibodies donkey anti-mouse (IRDye 800CW, 926–32212, LI-COR), anti-rabbit (IRDye 680CW, 926–68073, LI-COR) or anti-goat (IRDye 800CW, 926–32214, LI-COR) secondary antibodies.

### Silver Staining

The gel was fixed in 30 % ethanol and 0.5 % acetic acid for a minimum of 1 h under shaking conditions. The gel was then rinsed with 20 % ethanol and Milli-Q water for at least 10 min each. The gel was sensitized with 0.02 % freshly prepared sodium thiosulphate for 1 min, rinsed twice with Milli-Q water for 20 sec and stained with freshly prepared 0.2 % silver nitrate for 30 min, all under shaking conditions. After that another rinsing step with Milli-Q water for 5–10 sec was performed before the silver stain was developed with a freshly made developing solution (0.03 % formaldehyde, 3 % sodium carbonate, and 0.001 % sodium thiosulfate) to the desired intensity. Development was stopped with 5 % Tris base and 2.5 % acetic acid. The silver stain was imaged with the ChemiDox XRS+ and stored at room temperature in Milli-Q water.

### Bacterial Surface Protein Extraction

*Borrelia* surface proteins were extracted with the Pierce^™^ Cell Surface Protein and Biotynilation Kit (A44390, Thermo Scientific) according to the manufacturer’s instructions. A liquid culture were used for the extraction, washed three times with PBS, the number of *Borrelia* was assessed, and 1 × 10^9^ *Borrelia*/reaction were incubated with 2.4 mg of Sulfo-NHS-SS-Biotin for 10 min. *Borrelia* were then washed 3x with ice-cold TBS and lysed with lysis buffer containing 1 % protease inhibitor. After mixing the sample, it was incubated for 30 min on ice and spun down. The surface proteins were isolated from the lysate with NeutrAvidin^™^ Agarose column and eluted with the elution buffer combined with 10 % DTT.

### Aβ Binding Assay

Expression of Borrelia molecule molecules including FhbA from *B. afzelii*, *B. duttonii*, *B. hermsii*, and *B. miyamotoi* has been previously described^10^. A microbial molecule and Aβ42 (Anaspec Inc.) were incubated in PBS with 0.025 % Dithiothreitol (DTT) (Sigma-Aldrich) at 3 μM and 28 μM concentrations, respectively, for 72 h at room temperature using ApoE3 as a positive control^20^. After 72 h, the samples were immunoblotted either under reducing or non-reducing conditions, while a parallel gel was silver stained.

### ELISA

To further study the binding of microbial molecules to Aβ42, 10 μg/mL of the microbial molecules in 50 mM sodium bicarbonate buffer (pH 9.6) were coated on 96-well plates (Thermo Fisher Scientific) overnight at 4 °C utilizing LPS (Sigma-Aldrich) and fatty acid free BSA (P6156, Biowest) as negative controls^20^. The wells were washed once with 1x PBS prior to being blocked with 3 % fatty acid free BSA in 1x PBS at room temperature for 2 h. After the blocking step, wells were washed once with 1x PBS again and incubated at 37 °C with 600 nM Aβ42 in 1x PBS for 1 h. The wells were washed three times with 1x PBS and incubated with 100 μL of 0.25 μg/mL of the beta amyloid recombinant rabbit monoclonal antibody (702254, Thermo Fisher Scientific) in 0.3 % fatty acid free BSA in 1x PBS at 37 °C for 1 h. After three washes with 1x PBS, the wells were incubated with 0.2 μg/mL of the secondary HRP-conjugated anti-rabbit goat antibody (PC2852–1197, PerkinElmer) in 0.3 % fatty acid free BSA in 1x PBS. After three washes with 1x PBS,100 μL of TMB (34028, Thermo Fisher Scientific) were applied to the wells. The reaction was stopped after 15 min at room temperature with the addition of 100 μL of 0.5 M sulfuric acid. The absorbance was read using a 450 nm filter with the Hidex sense microplate reader (Hidexx Oy). The presence of soluble Aβ42 was done according to the manufacturer’s instructions using 1:2 dilutions (290–62601, Human β Amyloid(1–42) ELISA Kit Wako, FUJIFILM Wako Pure Chemical Corporation).

### Viability Assay with synthetic Aβ42 and *Borrelia* spp.

*Borrelia* spp. were washed thrice at 3000 × g for 5 min and diluted to 2 – 2.5 × 10^5^ *Borrelia*/mL in Hanks’ balanced salt solution (HBSS) (14175046, Gibco, Waltham, MA, USA). They were incubated in polypropylene tubes in the presence of 2 μM Aβ42 at 30 °C and 5 % CO_2_ for 1 hour ^20^. Half of the culture was used for immunoblotting, while BSK-H medium was added in a 1:4 dilution to the other half of the culture and Borrelia spp. were counted in the following two days.

### Viability Assay with ReN cell supernatant

*Borrelia* spp. were washed thrice at 3000 × g for 5 min and diluted to 1 × 10^6^ *Borrelia*/mL in different 3D ReN cell supernatants and Differentiation medium aged nine weeks. They were incubated in polypropylene tubes at 35 °C and 5 % CO_2_. The viability was counted at 0 h and 24 h. Of each culture 150 μL were used for immunoblotting.

### Microglial phagocytosis assay

A concentration of 1 × 10^9^ *Borrelia* spp. were labelled with 0.9 mM Alexa Fluor 568 C5 Maleimide (A20341, Thermo Fisher Scientific) by incubating them for 2 h at 22 °C, protected from the light. The *Borrelia* spp. were washed with 1x PBS until the supernatant was clear. The fluorescence was checked via the Olympus fluorescent microscope equipped with the 575 nm filter set. The labelled Borrelia spp. were aliquoted in 20 % glycerol in 1x PBS and stored at −80 °C.

Human immortalized microglia SV40 cells were cultured in Dulbecco’s Modified Eagle’s Medium (DMEM) with GlutaMax 4.5 g/L glucose (BE12–614F, Gibco) supplemented with 10 % fetal bovine serum (A5256801, Gibco), and with 5 % penicillin-streptomycin mix (ECB3001D, BioNordika Oy) at 37 °C in a T-75 cell culture flask (Greiner bio-one). The T-75 cell culture flask was coated with 42 μg/mL of rat tail type I collagen (PO 45202, Gibco) in 20 mM of sterile acetic acid (Sigma-Aldrich). The microglia were split every second day 1:10 by washing them first once with 5 mL of pre-warmed DPBS (14190144, Gibco) and detaching them with 1 mL of pre-warmed trypsin (25200056, Gibco) at 37 °C for 5 minutes. The trypsin was neutralized with 9 mL of DMEM. Cells were counted with the TC20TM automated cell counter (Bio-Rad).

Microglia SV40 cells were seeded in 5×10^4^ cells/mL in a coated 24-well flat bottom plate (Thermo Fisher Scientific) in triplicates for each experimental condition. Microglia SV40 cells were grown at 37 °C overnight. Maleimide-labelled *Borrelia* spp. were diluted and incubated in 1x PBS with 2 μM of Aβ42. The reaction was incubated at 30 °C in low-binding tubes (Eppendorf) at 400 rpm for 1 hour. *Borrelia* were diluted to 1×10^5^ bacteria/mL and added to the microglia that were spun down at 100 × g for 5 minutes and incubated at 37 °C in 5 % CO_2_ for either 2 h or 24 h. After the incubation, the supernatant was saved and counted, and the samples were washed thrice with 1x PBS, and imaged via the Axio Observer.z1 / 7 fluorescent microscope (Carl Zeiss Group) equipped with the LD Plan-Neofluar 20x/0.4 Korr M27 objective utilizing the excitation wavelength of 540 – 552 nm and bright field. The image resolution was 692 × 520 nm.

### Infection of 3D cultures

We used ReNcell^®^ VM Human Neural Progenitor Cell Line > genetically modified human neural progenitor cells (hNPCs) with familial AD mutations ^22^. Briefly, ReN cells, G2B2 and A5, were cultured in flasks precoated with 1:100 dilution of ice cold Matrigel (356234, Corning) in ice-cold DMEM/F12 medium (11320–033, Gibco). T75 flasks were coated with 8mL of Matrigel solution and incubated for 1 hour at room temperature. Media was aspirated and flasks were placed in 4 °C wrapped in saran wrap. Precoated flasks were used within 1 week. Confluent cells were washed with 5ml DPBS and detached with 2ml of warmed Accutase (A11105–01, Stemcell Technologies) at 37 °C incubator for 3–5 min. Cells were suspended in 4 ml of pre-warmed differentiation media (2 μg/mL heparin (07980, Stemcell Technologies), 1x B27 (17504–044, Gibco), 1 % penicillin-streptomycin mix, 1 % Amphotericin B (30–003-CF, Corning), DMEM/F12) and spun down at 300 × g for 5 min. A 24-well plate with 0.5 million cells per well was made with cold expansion medium (differentiation medium, 20 ng/mL EGF (E9644, Sigma-Aldrich), 20 ng/mL bFGF (03–002, Stemgent). The final volume of cells mixture was diluted with a 1:11 Matrigel. Pipette tip was chilled with cold medium before plating half with G2B2 and A5 and placed into 37 °C overnight. Next morning, media was replenished to differentiation medium yielding 1 million cells/ml. Media changes occurred twice a week until cells were differentiated at week 9–12. Before infection media was discarded and fresh media without antibiotics was added to each well.

For infection, *Borrelia* bacteria were cultured in BSK-H media and the number of bacteria in ml was counted under phase contrast objective using 200 × magnification. Bacteria were washed twice with DPBS and diluted in differential media in a concentration of MOI 10 (MOI = multiplicity of infection = 10 = 10 bact/cells in the culture) and incubated for 2 h at 37 °C 5% CO_2_. After incubation supernatant was collected for electron microscopy. The supernatant was centrifuged at 17,000 × g for 3 minutes fixed with 4% paraformaldehyde, washed with PBS and stored at 4 °C before Transmission electron microscopy (TEM).

For TEM a 10ul droplet of the fixed suspension was added to Formvar carbon-coated copper grids (FCF100-Cu, Electron Microscopy Sciences) and subject to a double-IGL protocol as follows before contrast staining with uranyl acetate. Immunogold Antibody staining; Grids were blocked with 1% BSA in PBS and then incubated with 1:1000 diluted anti-Amyloid antibody in blocking buffer. The grids were washed three times with PBS and incubated with goat anti-mouse IgG antibody covalently linked to nanogold particles. After three PBS washes and four washes with water, specimens were fixed with 1% glutaraldehyde. Samples were washed with water, stained with uranyl acetate, and then viewed using a JEM-1011 transmission electron microscope (JEOL Institute).

Supernatants of the 3D cultures were stored in the presence of 10 mM EDTA for WB to detect FH, C3b, Aβ and β-actin using goat anti-factor H (341276, Sigma-Aldrich), rabbit anti-C3c (BS6916R, Bioss Antibodies), rabbit anti-Aβ (700254, Thermo Fisher Scientific) and mouse anti-β-actin antibodies (A2228, Sigma-Aldrich) and corresponding secondary antibodies IRDye donkey anti-goat (926–68074, LI-COR), anti-rabbit (926–32213 or 926–68073, LI-COR) or anti-mouse antibodies (926–32212, LI-COR) and for Aβ and cytokine panel ELISAs (MSD V-PLEX Cytokine Panel). Bacteria in the supernatant was pelleted by centrifugation and washed with PBS to detect bacteria bound FH/C3b or unbound Aβ oligomers by WB.

The cells in the 3D culture were washed with ice cold DPBS and the cell-Matrigel suspension was scarped from the well with P1000 tip using ice cold PBS. The sample was spun at 8000 RPM for 2 min. at 4 °C and supernatant was removed. Pellet was dissolved in 40 ul 1% (wt/vol) sarkosyl and resuspended by vortexing 2 × 30 sec. After incubation for 30 min on ice the sample was centrifuged at 13300 × g for 10 min at 4 °C. Supernatant was collected and stored at −20 °C for WB.

Microglia were differentiated from induced pluripotent stem cells (iPSCs) ^42^. Briefly, cells were grown on 1:100 Matrigel coated 10 mL dishes in mTeSR plus basal media (100–0276, Stemcell Technologies) with 1 % penicillin-streptomycin mix until 70–80 % confluency. Cells were differentiated to hematopoietic stem cells using the differentiation kit (05310, Stemcell Technologies) according to the manufacturer’s instructions. From day 12, the cells were harvested and differentiated to microglia, seeding 20,000–25,000 HSCs/cm^2^. Cells were cultured in iMGL basal media (DMEM/F12, 2x B27, 0.5x N2 (17502–048, Gibco), 1x Glutamax (35050038, Gibco), 1x NEAA (12084947, Gibco), 400 μM monothioglycerol (M6145–25ML, Merck Life Science), 5 μg/mL insulin (I9278, Merck Life Science), 2x Insulin-transferrin-selenium (41400045, Gibco), 1 % penicillin-streptomycin mix, 0.05 % BSA (A1595, Merck Life Science) supplemented with 100 ng/mL IL-34 (17850433, Peprotech), 100 nM IDE1 (1164899, biogems), and 25 ng/mL M-CSF (PHC9504, Gibco). Fresh medium was added every second day. On days 12 and 25 cells in the supernatant were pelleted by centrifugation and added back to the plate with fresh media. From day 25, 100 ng/mL CD200 (A42571, Gibco) and 100 ng/mL CX3CL1 (17802893, Gibco) were added until cells were fully matured on day 27. Cells were maintained 2 weeks at maximum. To prepare 3D tetracultures, differentiated microglia were added to the 3D triculture and incubated for six days prior infection.

### Immunofluorescence Staining of 3D cultures

Cells were fixed with 4% paraformaldehyde (in PBS) at room temperature for 24 h (min. 4 h) and blocked with 4% BSA in 1× Tris-buffered Saline (TBS) with 0.1% (v/v) Tween-20 (TBS-T) for an additional 24 h at 4 °C. After washing with 1 × TBS-T once cells were permeabilized with a buffer containing 4% BSA and 0.5% Triton X-100 in TBS-T for 45 min at room temperature. After brief washing with 1× TBS-T, primary antibodies were added in a buffer containing 50 mM Tris-Cl (pH 7.4), 0.1% Tween-20, 4% BSA, 0.1% gelatin and 0.3 M glycine and incubate at 4 °C for 24. Antibodies that were used in different combinations are: goat anti-factor H, mouse anti-Aβ 6E10 (803015, Biolegend), rabbit anti-C3c rabbit anti-GFAP (ab16997, Abcam), rabbit anti-Borrelia burgdorferi (ABX48–1KC, Merck Millipore), goat Iba (ab5076, Abcam). The cells were washed three times with 1×TBS-T and incubated with Alexa Fluor secondary antibodies for 3.5 h at room temperature (AlexaFluor 405/568/647 donkey anti-mouse (A32787), -rabbit (A10042) and -goat (A48259) secondary antibodies (Invitrogen), 1:400 in different combinations). The cells were washed three times with 1×TBS-T and stored in TBS at 4°C. Fluorescence images were captured by Nikon C2s confocal laser scanning microscope (Nikon Instruments Inc.).

### Infection of BBB and 3D BBB cultures

LNB (*B. burgdorferi*) and RF (*B. hermsii*) were grown in BSK-H media at 37 °C 5% CO2 and washed twice with DPBS. A total amount of 3 × 10^7^ bacteria in a 200 μl volume of DPBS was labeled with 1:100 dilution of anti-Borrelia antibody (ABX48–1KC) for 30 minutes at +37 in a shaking incubator (250 rpm). After washing twice with DPBS the bacteria were labeled with 1:100 diluted 568 anti-rabbit antibody (A10042, Invitrogen) for BBB or 647 anti-rabbit antibody (A31573, Invitrogen) for 3D+BBB cultures in a 200 μl volume of BSK-H for 30 minutes at 37 °C in a shaking incubator (250 rpm). After incubation the bacteria were washed twice with BSK-H and the number of bacteria were counted by dark field microscopy. The BBB and 3DBBB cultures were prepared according to Pavlou, G. et al ^21^. BBB cultures washed three times with antibiotic LifeFactors^™^ mVasculife^®^ VEGF media (LS-1020 including 25% of suggested amount of Heparin, rh FGF-b (LS-1002, Lifeline), Ascorbic acid (LS-1005, Lifeline), Hydrocortisone (LS-1007, Lifeline), FBS (LS-1012, Lifeline), L-glutamine (LS-1013, Lifeline) or incubated overnight (3DBBB) with the media before bacterial infection. A total amount of 5 × 10^6^ labeled bacteria in VEGF media were infused to the 3D BBB cultures when the interstitial flow was removed. The media were replaced in the cultures after 1 h incubation and imaged after 1h using Nikon eclipse Ti confocal microcopy. The interstitial flow generated by syringes were placed back to the cultures and imaged again after 24 hours. Images were processed using ImageJ, Imaris analysis software, and ICY. After imaging, the culture supernatants were collected for detecting Borrelia proteins by WB. and for cytokine analysis (MSD V-PLEX Cytokine Panel) the supernatant was stored at −80 °C for cytokine analysis.

### Statistics

The CSF MS data was analyzed by IPA Qiagen software using cutoffs of Expr Log Ratio <−0.8 >0.8, and Expr p-value <0.01 and QIAGEN IPA Interpret program. Immunofluorescence data was analyzed using Fiji Image J. The Shapiro-Wilk test was used for normality testing. One-way ANOVA with Dunnett’s or Kruskal-Wallis test was used for multiple comparisons of unequally distributed samples while Tukey’s multiple comparisons were performed for sample sets with equal variances. Mann-Whitney U-test was used for pairwise comparisons. Statistics were calculated using SPSS version 29.0.2.0

## Supplementary Material

Supplementary Files

This is a list of supplementary files associated with this preprint. Click to download.

• ExtendeddataexcelfileS1.xlsx

• ExtendeddataFigures.pdf

• ExtendedDataFigureLegends.docx

**Supplementary information** files are included in this manuscript.
